# Clinical Application of Whole Exome Sequencing for Monogenic Disorders in PICU of China

**DOI:** 10.3389/fgene.2021.677699

**Published:** 2021-09-01

**Authors:** Yingchao Liu, Chanjuan Hao, Kechun Li, Xuyun Hu, Hengmiao Gao, Jiansheng Zeng, Ruolan Guo, Jun Liu, Jun Guo, Zheng Li, Zhan Qi, Xinlei Jia, Wei Li, Suyun Qian

**Affiliations:** ^1^Pediatric Intensive Care Unit, Beijing Children’s Hospital, Capital Medical University, National Center for Children’s Health, Beijing, China; ^2^Research Unit of Critical Infection in Children, Chinese Academy of Medical Sciences (2019RU016), Beijing, China; ^3^Beijing Key Laboratory for Genetics of Birth Defects, Beijing Pediatric Research Institute, MOE Key Laboratory of Major Diseases in Children, Beijing Children’s Hospital, Capital Medical University, National Center for Children’s Health, Beijing, China

**Keywords:** whole exome sequence, pediatric intensive care unit, monogenic disorders, clinical application, effective

## Abstract

**Objectives:**

Whole exome sequencing (WES) has been widely used to detect genetic disorders in critically ill children. Relevant data are lacking in pediatric intensive care units (PICUs) of China. This study aimed to investigate the spectrum of monogenic disorders, the diagnostic yield and clinical utility of WES from a PICU in a large children’s hospital of China.

**Methods:**

From July 2017 to February 2020, WES was performed in 169 critically ill children with suspected monogenic diseases in the PICU of Beijing Children’s Hospital. The clinical features, human phenotype ontology (HPO) terms, and assessment of clinical impact were analyzed.

**Results:**

The media age of the enrolled children was 10.5 months (range, 1 month to 14.8 years). After WES, a total of 43 patients (25%) were diagnosed with monogenic disorders. The most common categories of diseases were metabolic disease (33%), neuromuscular disease (19%), and multiple deformities (14%). The diagnosis yield of children with “metabolism/homeostasis disorder” and “growth delay” or “ocular anomalies” was higher than that of children without these features. In addition, the diagnosis rate increased when more features were observed in children. The results of WES had an impact on the treatment for 30 cases (70%): (1) change of treatment (*n* = 11), (2) disease monitoring initiation (*n* = 18), (3) other systemic evaluation (*n* = 3), (4) family intervention (*n* = 2), and (5) rehabilitation and redirection of care toward palliative care (*n* = 12).

**Conclusion:**

WES can be used as an effective diagnostic tool in the PICU of China and has an important impact on the treatment of patients with suspected monogenic conditions.

## Introduction

Congenital genetic disease is frequent in pediatric intensive care units (PICUs) (French et al., 2019). At present, almost 7,000 monogenic diseases have been listed on the OMIM website^1^, and this number is growing at a rate of approximately 30–50 per month. Children with monogenic diseases who are admitted to PICU are often in critical condition and accompanied by multiple organ dysfunction (Sanford et al., 2019). Considering the genetic and clinical heterogeneity of monogenic disease, critical physicians cannot provide a rapid and clear diagnosis and make a targeted treatment plan in time. The condition of the patient may continue to progress, increasing the risk of death. Cunniff et al. (1995) reported that the mortality rate of children admitted to the hospital due to birth defects and genetic diseases was 4.5 times higher than that of children admitted for other reasons. Therefore, clinicians must diagnose critically ill children with suspected monogenic diseases in time to provide them with timely interventions, improve prognosis, reduce mortality, and minimize the burden on families and the national health system.

Next-generation sequencing provides a new strategy for diagnosing genetic disorders. In recent years, an increasing number of studies have shown that whole exome sequencing (WES) has a unique value in the diagnosis of monogenic diseases. WES could be used as a first-tier test for children with suspected monogenic disorders (Stark et al., 2016; Hu et al., 2018). More than 20% of patients could be diagnosed using WES (Yang et al., 2013; Meng et al., 2017; French et al., 2019). Farnaes et al. (2018) reported that WES could reduce mortality and hospitalization costs. This may be due to the fact that after obtaining a definite diagnosis, the best clinical care can be provided for the affected children, promoting the transition from empirical treatment to definite treatment of identified diseases where feasible.

Previous studies of WES mainly included infants in neonatal intensive care units (NICUs) (Meng et al., 2017; Farnaes et al., 2018; Petrikin et al., 2018; Swaggart et al., 2019; Gubbels et al., 2020). However, studies about PICUs either had a relatively small number of cases or were reported jointly with NICUs (Mestek-Boukhibar et al., 2018; French et al., 2019; Sanford et al., 2019; Australian Genomics Health Alliance Acute Care et al., 2020; Wang et al., 2020a). The present study focused on the clinical application of WES in critically ill patients in the PICU. The diagnostic yield, the monogenic disease spectrum of patients, and effect of WES on clinical management were systematically analyzed for the first time in a PICU of China.

## Materials and Methods

### Study Design and Clinical Samples

We retrospectively analyzed the demographics and clinical characteristics of critically ill patients in the PICU who received WES, the diagnostic yield of WES, and the clinical management of diagnosed patients.

**FIGURE 1:**
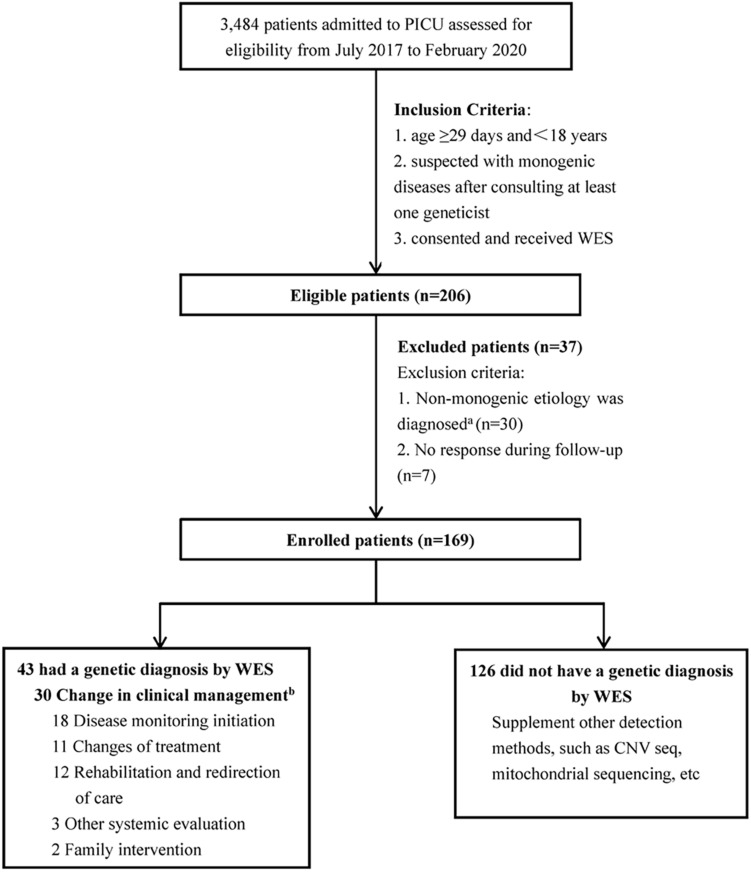
Flow diagram of the PICU patients who were enrolled, received genetic diagnosis by WES, and clinical management. ^a^The etiology of thirty patients was non-monogenic diseases, including: 19 acute necrotizing encephalopathy; 7 febrile infection-related epilepsy syndrome; 1 acute lymphoblastic leukemia; 1 poisoning; 1 heat stroke; 1 hemophagocytic syndrome. ^b^Some patients had changed the clinical management more than 1 category.

From July 2017 to February 2020, 3,484 patients were admitted to the PICU of Beijing Children’s Hospital. A total of 169 patients who met the inclusion criteria were enrolled in this study (Figure 1).

The inclusion criteria were: (1) age ≥29 days and <18 years, (2) suspected with monogenic diseases after consulting at least one geneticist, and (3) consented and received WES (trio-WES or proband WES). The exclusion criteria were as follows: (1) diagnosed with non-monogenic etiology and (2) no response during follow-up. This study was approved by the institutional review board of Beijing Children’s Hospital (No. 2020-Z-098).

### WES and Data Analysis

DNA was extracted from EDTA blood samples obtained from the probands and their parents (when available) using the Gentra Puregene Blood Kit (QIAGEN, Hilden, Germany). The SureSelect Human All Exon Kit V6 (Agilent Technologies, Santa Clara, United States) was used for whole exome capture. The target regions were sequenced on NovaSeq 6000 (Illumina, San Diego, United States). Raw reads were filtered using FastQC to remove low-quality reads. Clean reads were mapped to the reference genome GRCh37. Quality control information included the following: average read depth of >100×, accurate mapping rate of >98%, base capture rate of >55%, 20× mean depth coverage rate of >96%, and duplication rate of <25%. Single nucleotide variants were annotated and filtered by TGex^2^. Variants with a frequency over 1% in the gnomAD, ESP, or 1000G databases were excluded. Variants that lacked segregation in family members were also filtered. Variants were classified following the American College of Medical Genetics and Genomics and the Association for Molecular Pathology interpretation standards and guidelines (Richards et al., 2015). The genetic results were reviewed by a multidisciplinary team which included geneticists, pediatric intensive care physicians, neurologists as required, and compared with the phenotypes, related biochemical tests, or imaging examinations of the patients to make the final diagnosis (Supplementary Table 1).

### Phenotype Analysis

The clinical features and the impact on patient prognosis were evaluated by reviewing electronic medical records. The clinical features were translated into human phenotype ontology (HPO) terms through the HPO website^3^. The top-level branching of HPO phenotypes was analyzed to ensure adequate counts of participants. The diagnostic rates of patients with and without HPO terms, the relationship between the phenotype complexity and expected diagnostic yield were analyzed.

### Assessment of Clinical Impact

The changes in clinical management were determined from the medical records and follow-up by telephone until June 2020. These changes were grouped into five categories: (1) changes of treatment, (2) disease monitoring initiation, (3) other systemic evaluation, (4) family intervention, and (5) rehabilitation and redirection of care toward palliative care.

### Statistical Analysis

All analyses were conducted in SPSS 22.0. Categorical variables were expressed as frequencies and percentages, continuous variables as median (interquartile range). Differences between groups were determined by Student *t*-test, Mann-Whitney *U* test, chi-square, if the expected counts were less than 5, Fisher’s exact test. *p*-values of less than 0.05 were considered to indicate statistical significance.

## Results

### General Information

**FIGURE 2:**
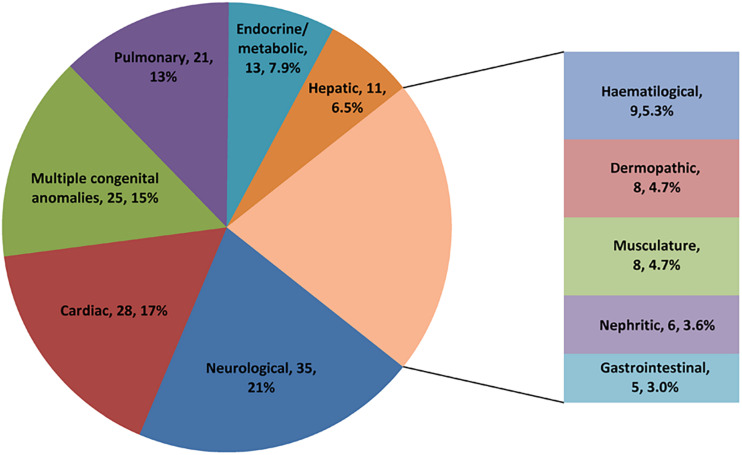
The composition of primary clinical manifestations of the 169 enrolled patients.

We enrolled for WES analysis 169 patients, 96 (57%) males, and 73 (43%) females. The ages of patients ranged from 1 month to 14.8 years, and the median age was 10.5 months (4 months, 47.5 months). The most common primary manifestation was neurological (21%), followed by cardiac (17%) and multiple congenital anomalies (15%) (Figure 2). The conditions of affected patients were severe with 82% of patients requiring mechanical ventilation and 62% of patients needing endotracheal intubation. In addition, more than 20% of patients received inotropic support or continuous renal replacement therapy. The mortality rate at discharge was 12%, and the mortality at 120 days after discharge was 32%.

### Diagnostic Yield of WES in PICU

Among the 169 enrolled patients, trio WES was performed on 160 (95%) patients, while proband-only WES was performed on the remaining nine (5.3%) patients on the basis of the availability of parental samples. Forty-three cases had monogenic disorders, and the diagnosis rate was 25%.

In the 43 diagnosed cases, 40 monogenic diseases were detected. The most common type was metabolic diseases (14/43, 33%), followed by neurological and muscular diseases (8/43, 19%), multiple malformations (6/43, 14%), cardiac diseases (4/43, 9.3%), immunodeficiency diseases (2/43, 4.7%), nephritic diseases (2/43, 4.7%), and other diseases (7/43, 16%) (Table 1 and Supplementary Table 1). *ATP7B*-related Wilson disease and *KMT2D*-related Kabuki syndrome were observed in 3 (7.0%) and 2 (4.7%) patients, respectively. The three children with Wilson disease had an initial symptom of acute liver failure, and then they were admitted to the PICU. Of all diagnosed patients, 20 (46%) were autosomal dominant (with 14 *de novo*, 2 inherited, and 4 inherited unknown), 21 (49%) were autosomal recessive (with 18 compound heterozygous and 3 homozygous), one was X-linked dominant (with *de novo*), and one was X-linked recessive (with maternal carrier).

**TABLE 1:** Brief clinical and genetic information of 43 patients with positive molecular diagnosis.

Case	Age	Sex	Phenotype	Gene	Variant	Inheritance Pattern	Zygosity	Disease	Clinical impact
004	2m	M	Congenital hypertrophy of left ventricle; Patent ductus arteriosus; Hypertrichosis; Coarse facial features	*ABCC9*	c.3203T > C (p.Leu1068Pro)	AD	*De novo* het	Cantu syndrome (OMIM[#]239850)	Cardiac surveillance
006	8y1m	F	Jaundice; Pigment gallstones; Splenomegaly; Non-spherocytic hemolytic anemia; Seizure; Loss of consciousness	*GPI*	c.637T > A (p.Phe213Ile); c.1614C > G (p.His538Gln)	AR	Compound het	Hemolytic anemia, non-spherocytic, due to glucose phosphate isomerase deficiency (OMIM[#]613470)	Splenectomy; Follow-up of hematology major regularly
012	3y	F	Renal insufficiency; Anemia	*ANKS6*	c.2420dupT (p.Thr808Aspfs*2); c.1973-1G > A	AR	Compound het	Nephronophthisis 16 (OMIM[#]615382	Redirection of care
017	4y6m	F	Lethargy; Coma; Seizures; Muscular hypotonia; Acute hyperammonemia; Global developmental delay	*MCCC1*	c.639 + 2T > A; c.1679dupA (p.Asn560Lysfs*10)	AR	Compound het	3-methylcrotonyl-CoA carboxylase 1 deficiency (OMIM[#]210200)	Rehabilitation; Protein-restricted diet; Biotin supplementation; Ammonia surveillance
026	5m	F	Pneumonia; Diarrhea; Severe combined immunodeficiency; Panhypogammaglobulinemia; Severe T lymphocytopenia; B lymphocytopenia	*RAG1*	c.1528G > T (p.Glu510*); c.2393A > G (p.His798Arg)	AR	Compound het	Severe combined immunodeficiency, autosomal recessive, T cell-negative, B cell-negative, NK cell-positive (OMIM[#]601457)	NA
027	3y6m	M	Failure to thrive; Global developmental delay; Seizure; Hypertrophic cardiomyopathy	*MAP2K1*	c.389A > G (p.Tyr130Cys)	AD	Het	Cardiofaciocutaneous syndrome 3 (OMIM[#]615279)	NA
032	3m	M	Weak cry; Premature birth; Intrauterine growth retardation; Small for gestational age; Limb muscle weakness; Failure to thrive; Tachypnea; Respiratory failure; Hyporeflexia; Global developmental delay; Camptodactyly of finger	*IGHMBP2*	c.1061-2A > G	AR	Homo	Spinal muscular atrophy, distal, autosomal recessive, 1 (OMIM[#]604320)	Redirection of care
034	13y10m	M	Hepatic steatosis; Visual impairment; Abnormality of lipid metabolism; Nystagmus; Global developmental delay; Hepatomegaly; Splenomegaly; Hypertriglyceridemia; Renal insufficiency; Type II diabetes mellitus; Hypertension; Pulmonary hypertension; Diabetes insipidus; Dilated cardiomyopathy	*ALMS1*	c.8532_8533insA (p.Leu2845Thrfs*3); c.9152_9153delCT (p.Cys3053Serfs*9)	AR	Compound het	Alstrom syndrome (OMIM[#]203800)	Redirection of care
041	3y10m	M	Hypertrophic cardiomyopathy; Muscular hypotonia; Dyspnea; Respiratory insufficiency due to muscle weakness; Myopathy; Arrhythmia	*GAA*	c.1082C > T (p.Pro361Leu); c.2051C > T (p.Pro684Leu)	AR	Compound het	Glycogen storage disease II(OMIM[#]232300)	Redirection of care; Sister diagnosed and started on enzyme replacement therapy
043	1y8m	M	Agammaglobulinemia; Pneumonia	*BTK*	c.1781G > A (p.Gly594Glu)	XLD	*De novo* hemi	Agammaglobulinemia, X-linked (OMIM[#]300755)	Immunoglobulin replacement therapy
050	1m	F	Coarctation of aorta; Hirsutism; Micrognathia	*KMT2D*	c.14113_14123del11(p.Ile4705Phefs*4)	AD	*De novo* het	Kabuki syndrome 1 (OMIM[#]147920)	Routine monitoring of aortic valve stenosis; Endocrinology and audiology evaluation
057	1y3m	F	Diarrhea; Enterocolitis; Perianal abscess; Pyoderma	*IL10RA*	c.301C > T (p.Arg101Trp); c.537G > A (p.Thr179Thr)	AR	Compound het	Inflammatory bowel disease 28, autosomal recessive (OMIM[#]613148)	NA
059	12y2m	M	Lethargy; Hyperammonemia; Vomiting; Hepatomegaly; Global developmental delay	*ASL*	c.91G > A (p.Asp31Asn); c.965_966insCAAAGACTT (p.Asn322_Lys323insLysAspPhe)	AR	Compound het	Argininosuccinic aciduria (OMIM[#]207900)	Protein-restricted diet; Arginine; Ammonia surveillance
065	2m	M	Feeding difficulties; Dystonia; EEG abnormality; Seizures; Horizontal nystagmus	*GABRB2*	c.909G > T (p.Lys303Asn)	AD	*De novo* het	Epileptic encephalopathy, infantile or early children,2 (OMIM[#]617829)	NA
075	8y4d	F	Jaundice; Hemolytic anemia; Hepatic failure; Kayser-Fleischer ring; Decreased serum ceruloplasmin; Increased urinary copper concentration	*ATP7B*	c.2333G > T (p.Arg778Leu)	AR	Homo	Wilson disease (OMIM[#]277900)	Penicillamine and Zinc Sulfate; Liver function and hemolysis surveillance
081	11m7d	M	Dilated cardiomyopathy; Left ventricular non-compaction cardiomyopathy; Cardiac arrest	*CASZ1*	c.2443_2459del GTGGGCACCCCCAGCCT (p.Val815Profs*14)	AD	*De novo* het	^a^CASTOR ZINC FINGER PROTEIN 1; CASZ1 (OMIM[*]609895)	NA
084	1y1m	F	Seizures; Global developmental delay; Microcephaly; Muscularhypotonia; Hepatomegaly	*IFIH1*	c.1852C > T (p.Arg618*)	AD	*De novo* het	Aicardi-Goutieres syndrome 7 (OMIM[#]615846)	Redirection of care
091	10m	F	Vomiting; Elevated hepatic transaminases; Metabolic acidosis	*HMGCS2*	c.1347_1351del AGCCT (p.Ala450Profs*7) c.1201G > T (p.Glu401*)	AR	Compound het	3-hydroxy-3-methylglutaryl-CoA synthase-2 deficiency (OMIM[#]605911)	Avoid prolonged hunger; Coenzyme Q10 and levocarnitine; Liver function and blood gas analysis surveillance
096	2m	F	Neutropenia; Thrombocytopenia; Lethargy; Hyperhomocystinemia; Methylmalonic acidemia	*MMACHC*	c.566_567insT (p.Ile190Tyrfs*13); c.609G > A (p.Trp203*)	AR	Compound het	Methylmalonic aciduria and homocystinuria, cblC type (OMIM[#]277400)	Redirection of care; Vitamin B6, Vitamin B12, levocarnitine, folic acid and betaine
103	5m	F	Microcephaly; Premature birth; Small for gestational age; Intrauterine growth retardation; Recurrent infections; Bilateral single transverse palmar creases; Global developmental delay	*RNU4ATAC*	n.16G > A (non-coding transcript variant-regulatory mutation); n.51G > A (non-coding transcript variant-regulatory mutation)	AR	Compound het	Roifman syndrome (OMIM[#]616651)	NA
105	5y11m	M	Pulmonary artery stenosis; Peripheral arterial stenosis; Supravalvular aortic stenosis	*ELN*	c.1621C > T (p.Arg541*)	AD	Het	Supravalvular aortic stenosis (OMIM[#]185500)	Redirection of care
106	2m	M	Webbed neck; Low-set ears; Hypertelorism Pulmonic stenosis	*KRAS*	c.173C > T (p.Thr58Ile)	AD	Het	Noonan syndrome 3 (OMIM[#]609942)	Routine monitoring of pulmonic; Regular follow-up of cardiac and neurology
109	2m	M	Type I diabetes mellitus; Diabetic ketoacidosis	*EIF2AK3*	c.641A > G (p.Tyr214Cys); c.12delC (p.Ile5Serfs*66)	AR	Compound het	Epiphyseal dysplasia, multiple, with early-onset diabetes mellitus (OMIM[#]226980)	Height, weight and growth evaluation
112	2y2m	F	Muscular hypotonia; Hyporeflexia; Motor delay; Distal muscle weakness; Infantile onset	*MPZ*	c.380G > A (p.Cys127Tyr)	AD	*De novo* het	Hypertrophic neuropathy of Dejerine-Sottas (OMIM[#]145900)	NA
113	2y5m	M	Motor delay; Neonatal hypotonia; Muscular hypotonia; Generalized muscle weakness; Infantile onset	*RYR1*	c.6721C > T (p.Arg2241*); c.5915A > T (p.Asn1972Ile)	AR	Compound het	Central core disease of muscle (OMIM[#]117000)	Redirection of care
120	6m	F	Infantile onset; Generalized myoclonic seizures; Status epilepticus; Epileptic encephalopathy	*SCN1A*	c.2758C > A (p.Arg920Ser)	AD	*De novo* het	Epileptic encephalopathy, early infantile, 6 (OMIM[#]607208)	NA
140	1m	F	Cyanosis; Bilateral tonic-clonic seizure with focal onset	*SCN2A*	c.4976C > T (p.Ala1659Val)	AD	*De novo* het	Seizures, benign familial infantile, 3(OMIM[#]607745)	Redirection of care
149	5y3m	M	Cryptorchidism; Hypospadias; Hypertelorism; Anal atresia; Pectus excavatum	*MID1*	c.1863_1879dupGAACTCCA TCCACCTCT (p.Tyr627*)	XLR	Inherited hemi (maternal carrier)	Opitz GBBB syndrome, type I (OMIM[#]300000)	NA
151	1m	M	Elevated hemoglobin A1c; Hyperglycemia; Diabetic ketoacidosis	*KCNJ11*	c.601C > T (p.Arg201Cys)	AD	*De novo* het	Diabetes mellitus, transient neonatal, 3(OMIM[#]610582)	NA
153	1m	M	Defect in the atrial septum; Ventricular septal defect; Muscular hypotonia; Macrotia; Hypoglycemia	*KMT2D*	c.5269C > T (p.Arg1757*)	AD	*De novo* het	Kabuki syndrome 1 (OMIM[#]147920)	Cardiac surveillance; Endocrinology and audiology evaluation
154	8m	M	Seizures; Lethargy; Global developmental delay; Hypoglycemia; Metabolic acidosis; Hepatomegaly; Medium chain dicarboxylic aciduria; Muscular hypotonia	*ACADM*	c.449_452delTGAC (p.Thr150Argfs*4); c.1010A > C (p.Tyr337Ser)	AR	Compound het	Acyl-CoA dehydrogenase, medium-chain, deficiency of (OMIM[#]201450)	Avoid prolonged hunger; Levocarnitine; Regular follow-up of neurology; Initiated a reproductive program
159	13y4m	F	Glioblastoma	*TP53*	c.375G > A (p.Thr125Thr)	AD	Inherited het (Paternal carrier)	Glioma susceptibility 1 (OMIM[#]137800)	NA
176	9y7d	M	Sinus bradycardia; Atrioventricular block; Sick sinus syndrome; Ventricular escape rhythms	*SCN5A*	c.4895G > A (p.Arg1632His)	AD	Inherited het (maternal carrier)	Sick sinus syndrome 1 (OMIM [#]608567)	Electrocardiogram and echocardiography surveillance regularly
177	5m	M	Psoriasis; Pustule Erythema	*IL36RN*	c.115 + 6T > C	AR	Homo	Psoriasis 14, pustular (OMIM[#]614204)	Follow-up of dermatological major
178	2y3m	M	Short stature	*P4HB*	c.236dupT (p.Leu79Phefs*11)	AD	*De novo* het	Cole-carpenter syndrome 1 (OMIM[#]112240)	NA
181	12y5m	M	Pectus excavatum; Tall stature; Dolichocephaly; Narrow face; Long face; Myopia; Decreased subcutaneous fat; Aortic root dilation	*FBN1*	c.1995C > A (p.Tyr665*)	AD	*De novo* het	Marfan syndrome (OMIM[#]154700)	Routine monitoring of aorta
186	2y8m	M	Lethargy; Seizures; Elevated hepatic transaminases; Hepatomegaly; Hyperammonemia; Myopathy	*CPT2*	c.1148T > A (p.Phe383Tyr); c.1749C > A (p.Asn583Lys)	AR	Compound het	Carnitine palmitoyltransferase II deficiency, infantile (OMIM[#]600649)	High-carbohydrate and low-fat diet; Levocarnitine; Regular monitoring of liver function, muscle enzyme, blood glucose, blood lipid and echocardiography
187	1y2m	F	Dilated cardiomyopathy	*TNNI3*	c.536A > G (p.Glu179Gly)	AD	*De novo* het	Cardiomyopathy, dilated, 1FF (OMIM[#]613286)	Cardiac surveillance
196	8y8m	F	Pheochromocytoma; Episodic hypertension; Renal artery stenosis; Elevated urinary norepinephrine; Proteinuria	*SDHB*	c.137G > A (p.Arg46Gln)	AD	Het	Pheochromocytoma (OMIM[#]171300)	NA
197	14y7m	F	Jaundice; Hemolytic anemia; Proteinuria; Hepatic failure; Kayser-Fleischer ring; Decreased serum ceruloplasmin; Increased urinary copper concentration	*ATP7B*	c.3836A > G (p.Asp1279Gly); c.3168delT (p.Leu1057Trpfs*64)	AR	Compound het	Wilson disease (OMIM[#]277900)	Penicillamine and zinc sulfate; Liver function and hemolysis surveillance
201	8y8m	F	Hepatic failure; Jaundice; Cholestatic liver disease; Decreased serum ceruloplasmin	*ATP7B*	c.2333G > T (p.Arg778Leu); c.2975C > T (p.Pro992Leu)	AR	Compound het	Wilson disease (OMIM[#]277900)	Redirection of care
204	2m26d	M	Elevated hepatic transaminases; Lactic acidosis; Hypoglycemia; Hyperuricemia; Hepatomegaly; Protuberant abdomen; Hypertriglyceridemia	G6PC	c.248G > A (p.Arg83His); c.310C > T (p.Gln104*)	AR	Compound het	Glycogen storage disease Ia (OMIM[#]232200)	Avoid prolonged hunger; Monitor blood glucose, height, weight; Endocrine follow-up
205	4m15d	M	Decreased serum complement factor H; Hematuria	*CFH*	c.3548G > T (p.Trp1183Leu)	AD	*De novo* het	Complement factor H deficiency (OMIM[#]609814)	Redirection of care

*^*a*^Gene description see Guo et al. (2019). NA, not available. AD, autosomal dominant; AR, autosomal recessive; Het, heterozygous; Hemi, hemizygous; Homo, homozygous; XLD, X-linked dominant; XLR, X-linked recessive.*

**FIGURE 3:**
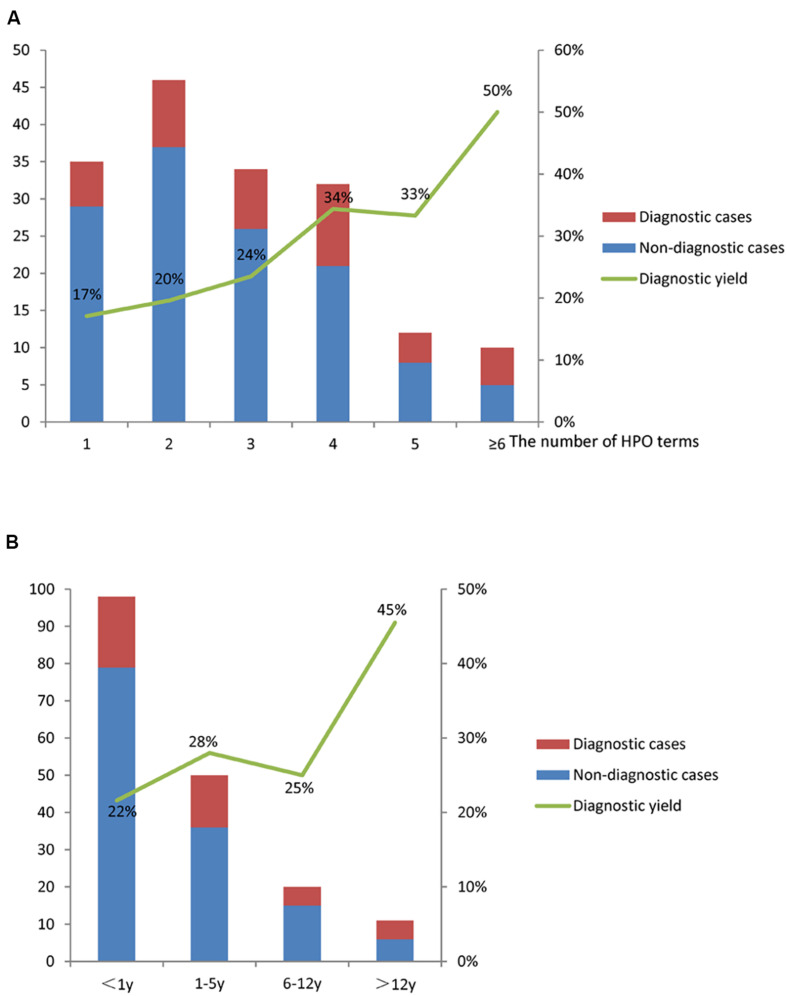
The diagnostic yield of WES in the PICU. **(A)** The diagnostic yield increased with the number of HPO terms; **(B)** the diagnostic yields for different age groups were different.

In this study, the diagnostic rates were compared among patients with different phenotypes represented by HPO terms. We found that specific clinical features were associated with a molecular diagnosis. For example, patients with neurological and cardiovascular anomalies received diagnosis rates higher than 30%. Patients with HPO terms of “Abnormality of metabolism/homeostasis” (HP: 0001939), “Growth abnormality” (HP: 0001507), and “Abnormality of the eye” (HP: 0000478) had a higher diagnostic yield than those without the terms (Table 2) (*P* < 0.05). Moreover, we observed the relationship between the complexity of patient’s phenotype (reflected by the number of HPO terms) and the expected diagnostic rate (Figure 3A). The diagnostic yield of cases with only one HPO term was 17%. When more than three HPO terms were given, the diagnostic rate will increase and exceed 30%. For HPO terms greater than 6, the diagnosis rate will reach as high as 50%. However, no statistical difference was observed in the diagnosis yield between children with different HPO terms (*P* = 0.24).

**TABLE 2:** Comparison of diagnostic rate by whole exome sequencing in groups with and without the phenotype.

HPO terms	Total (%)	Diagnostic rate in	Diagnostic rate in patients	*P-*value^*a*^
		patients with the term (%)	without the term (%)	
Abnormality of the eye (HP:0000478)	11(6.5)	7/11 (64)	36/158 (23)	**0.006**
Abnormality of metabolism/homeostasis (HP:0001939)	35 (21)	15/35 (43)	28/134 (21)	**0.007**
Growth abnormality (HP:0001507)	11 (6.5)	6/11 (55)	37/158 (23)	**0.03**
Abnormality of head and neck (HP:0000152)	12 (7.1)	6/12 (50)	37/157 (24)	0.06
Abnormality of connective tissue (HP:0003549)	4 (2.4)	0/4 (0)	43/165 (26)	0.12
Abnormality of the ear (HP:0000598)	3 (1.8)	2/3 (67)	41/166 (25)	0.16^*a*^
Abnormality of the musculature (HP:0003011)	28 (17)	10/28 (36)	33/141 (23)	0.17
Abnormality of the endocrine system (HP:0000818)	12 (7.1)	5/12 (42)	38/157 (24)	0.20
Abnormality of the integument (HP:0001574)	29 (17)	10/29 (34)	33/140 (24)	0.22
Abnormality of voice (HP:0001608)	1 (0.6)	1/1 (100)	42/168 (25)	0.25^a^
Abnormality of the respiratory system (HP:0002086)	38 (22)	7/38 (18)	36/131 (27)	0.26
Abnormality of digestive system (HP:0025031)	49 (29)	15/49 (31)	28/120 (23)	0.32
Abnormality of the cardiovascular system (HP:0001626)	58 (34)	13/58 (22)	30/111 (27)	0.51
Abnormality of the skeletal system (HP:0000924)	16 (9.5)	5/16 (31)	38/153 (25)	0.58
Abnormality of blood and blood-forming tissues (HP:0001871)	31 (18)	9/31 (29)	34/138 (25)	0.61
Abnormality of the nervous system (HP:0000707)	72 (43)	17/72 (24)	26/97 (27)	0.64
Abnormality of limbs (HP:0040064)	6 (3.6)	2/6 (33)	41/163 (25)	0.66
Neoplasm (HP:0002664)	7 (4.1)	2/7 (29)	41/162 (25)	0.85
Abnormality of the genitourinary system (HP:0000119)	23 (14)	6/23 (26)	37/146 (25)	0.94
Abnormality of the immune system (HP:0002715)	35 (21)	9/35 (26)	34/134 (25)	0.97
Abnormality of prenatal development or birth (HP:0001197)	8 (4.7)	2/8 (25)	41/161 (25)	0.98

*^*a*^Fisher’s exact test. The bold values are less than 0.05, which indicate statistical significance.*

The diagnostic yield of patients aged more than 12 years was the highest (45%), and that of patients aged less than 1 year was relatively low (22%) (Figure 3B). No statistical difference was observed in the diagnosis rate between children of different age groups (*P* = 0.36).

The media turnaround time of WES in diagnosed patients was 11 days, of which 44% of patients received definite diagnosis before discharge.

### Clinical Impact of WES Diagnosis

In this study, 30 patients (70%) were affected by the clinical management. We evaluated the impact of WES on clinical treatment through the following aspects (Table 1 and Supplementary Table 1).

#### Changes of Treatment

Treatment changes affected 11 patients. Due to infection-induced “hemolytic crisis” with seizure and impairment of consciousness, case 006 was admitted to the PICU. In addition, the child was hospitalized many times because of repeated jaundice. This patient was diagnosed as *GPI*-related non-spherocytic hemolytic anemia (OMIM: 613470) through WES and discharged from the hospital after laparoscopic cholecystectomy and splenectomy. Her condition was normal, and her jaundice was significantly alleviated.

Moreover, some metabolic diseases required diet therapy in addition to drug treatment. For example, a patient (case 059) with argininosuccinic aciduria (OMIM: 207900) received a protein-restricted diet, another child (case 186) with carnitine palmitoyltransferase II deficiency (OMIM: 600649) needed a high-carbohydrate and low-fat diet, while other children should avoid prolonged hunger.

#### Disease Monitoring Initiation

Eighteen patients underwent further disease monitoring after diagnosis. For example, regular electrocardiogram monitoring and echocardiography surveillance were required for case 176 with sick sinus syndrome to monitor for arrhythmias. Liver function and blood gas monitoring was critical for case 91 with 3-hydroxy-3-methylglutaryl-CoA synthase-2 deficiency to understand the metabolic state of the body and provide timely intervention. Routine neurological examinations were required for patients with brain damage.

#### Other Systemic Evaluation

After the diagnosis of WES, three patients were subjected to other systemic evaluation: cases 050 and 153 had *KMT2D*-related Kabuki syndrome 1 (OMIM: 147920) and underwent endocrinology and audiology evaluations. Case 109 had *EIF2AK3*-related multiple epiphyseal dysplasia with early-onset diabetes mellitus (OMIM: 226980). In addition to ongoing diabetes treatment, height, weight, growth, and development were evaluated and monitored.

#### Family Intervention

In addition to the effect on medical services, WES also had a potential influence on family members. Case 041 was diagnosed with glycogen storage disease II (OMIM: 232300). The child’s 9-year-old sister had normal activity endurance but could not tolerate activities such as dancing and running. She underwent relevant genetic testing and was subsequently diagnosed. Fortunately, she was started on enzyme replacement therapy, and lives a normal life at present. Another family initiated a reproductive program through genetic counseling.

#### Rehabilitation and Redirection of Care Toward Palliative Care

Considering the severity and poor prognosis of the diseases, parents may make different decisions after the diagnosis of WES. Case 017 had 3-methylcrotonyl-CoA carboxylase 1 deficiency (OMIM: 210200). After treatment in the PICU, the child’s vital signs gradually stabilized, but consciousness did not return. The parents chose to leave the PICU for rehabilitation training and accompany the child after diagnosis. The child is now conscious and able to walk. However, 11 patients’ family members chose to stop invasive surgery or life support equipment to alleviate the pain of their children. Most of the children suffered from severe disorders or life-limiting conditions.

## Discussion

WES has been increasingly used to diagnose genetic diseases. Previous studies on WES mainly focused on NICUs (Smith et al., 2015; Willig et al., 2015; Wang et al., 2020b). In the present study, we first reported the use of WES in a relatively large number of critically ill children in a PICU from China. The age of patients in this study ranged from 1 month to 14.8 years, with an average age of 2.8 years, which was older than those in previous similar studies (Mestek-Boukhibar et al., 2018; French et al., 2019; Australian Genomics Health Alliance Acute Care et al., 2020; Wang et al., 2020a). The most common presentation was neurological (21%), cardiac (17%), and multiple congenital anomalies (15%), which were similar to those in previous studies (Farnaes et al., 2018; Wang et al., 2020a).

The diagnosis yield varied with different phenotypes, and increased with the phenotypic complexity. Stark et al. (2016) reported that infants with characteristics of strongly suggestive monogenic diseases, such as neurometabolic status and skeletal dysplasia, received WES, and the diagnosis yield was 57%. In the present study, the indication for clinical WES that was assessed by HPO phenotype analysis as having high diagnosis value included (1) “abnormality of metabolism/homeostasis,” such as metabolic acidosis; (2) “growth abnormality,” such as failure to thrive; and (3) “abnormality of the eye,” such as hypertelorism. Therefore, the strict screening of phenotypes can be used to improve the diagnosis yield. However, a previous study had shown that restricting the recruitment to these specific phenotypes would miss many important diagnoses (French et al., 2019). In the future work, more stringent screening should be carried out on the conditions for joining the group to improve the diagnosis rate. Interestingly, Trujillano et al. (2017) reported that complex phenotypes including several symptoms tend to have a higher diagnostic yield, which is consistent with our findings. This might suggest that patients’ phenotypes with several specific symptoms have a higher probability to be explained by monogenic reasons. Therefore, we recommend physicians who request WES to include as many symptoms as possible in their clinical description, but to mark those symptoms that seem to be specific to a particular patient. WES can find the complex manifestations of monogenic diseases. With its wide application in clinical practice, it can be used to easily diagnose patients with overlapped symptoms and mixed phenotypes.

Combined with other detection methods or information supplementation, the diagnosis yield could be improved. Hu et al. (2020) reported that through parallel tests of WES and copy number variant sequencing, the diagnosis yield could be increased from 37 to 53%. In the present study, for patients who were strongly suspected of monogenic diseases but could not be diagnosed after WES, pediatric intensive care physicians and geneticists supplemented the patient’s phenotype or made suggestions to add other detection methods (e.g., copy number variant sequencing, mitochondrial sequencing) through clinical/laboratory rounds and online communication. In this way, two patients were diagnosed. Case 148 with muscular hypotonia and progressive dyspnea received a diagnosis by mitochondrial genome sequencing. He was diagnosed with Leigh syndrome (OMIM: 256000). Case 206 with development delay was diagnosed through copy number variant sequencing (8q24.3 dup 3.6 Mb; 18q22.2-q23 del 9.9 Mb). In the future work, we will continue to strengthen clinical–laboratory interaction so as to improve the efficiency of diagnosis.

For the uncertain significance variants in this study, the following methods usually were taken to help improve the judgment result: (1) Supplement related tests, such as case 006, the final diagnosis was confirmed through supplementation of glucose phosphate isomerase activity test; (2) Test relatives of the proband with the same symptoms, such as case 041, the WES test was performed on the sister of the patient with the same symptoms, and there was a same variant, then the final diagnosis was given; (3) Perform functional verification (not covered in this article).

In addition, the diagnosis yield was also affected by socioeconomic and age factors. In this study, the diagnosis yield of children aged >12 years was the highest (45%), whereas that of children under 1 year was 22%, which was lower than that previously reported (Meng et al., 2017; Wang et al., 2020a). This may be due to the fact that more than half of the enrolled patients in this study were less than 1 year old and were treated in the PICU at the first onset, and their phenotypes were often atypical, mostly congenital heart disease and convulsions. Their parents were willing to find the etiology of the disease through relevant tests. Therefore, in this age group, the percentage of patients who received WES was high, but the diagnosis yield was relatively low. On the other hand, children who were >12 years old often had obvious phenotypes when they were hospitalized and experienced the diagnostic odyssey outside. Thus, they had a higher chance to be diagnosed by WES.

Among the critically ill children, 48–81% of the diagnosed children had changes in clinical management (Willig et al., 2015; Meng et al., 2017; Wu et al., 2019; Wang et al., 2020a). In this study, the diagnosis of WES affected 30 cases (70%) in various aspects. First, WES had an impact on medical treatment: (1) **Implement any method, including drugs, surgery, and diet, while avoiding other ineffective or potentially harmful treatments**. For example, in case 006 who was diagnosed with *GPI*-related non-spherocytic hemolytic anemia, she was hospitalized many times in the past because of repeated jaundice. Splenectomy was performed after definite diagnosis. The available data showed that splenectomy was a common and effective treatment (Beutler et al., 1974; Fermo et al., 2019). Thereafter, the need for blood transfusions could be significantly reduced, and the quality of life of patients could be greatly improved. Although the prognosis is serious for some metabolic diseases, earlier diagnosis could lead to better results through diet control (Erez et al., 2011). (2) **Disease surveillance and evaluation of the system involved**. Single gene diseases often cause multi-system damage. After a clear diagnosis, through targeted disease surveillance, the affected organs could be identified. Once problems occur, interventions should be made in advance to achieve the purpose of long-term survival. For example, case 176 was diagnosed with sick sinus syndrome 1, and electrocardiogram and echocardiography should be monitored regularly. The genetic diagnosis also has impacts on the family: (1) **Change of attitude to treatment**. For patients with definite diagnosis, if the disease could be treated (including surgery, medicine, diet), parents are more confident to cooperate with doctors for treatment and regular re-examination. Even for patients with unclear diagnosis, some genetic diseases may be excluded. Thus, parents are urged to actively seek further diagnosis and treatment. In our study, roughly 20% of parents chose positive treatment. (2) **Withdrawing treatment**. For serious diseases that cannot be cured, parents have also found the causes of their children’s illness. Some parents could consider earlier overall hospice care to alleviate their children’s pain in the final stages of life and accompany them through the last days of their lives (Oberender and Tibballs, 2011). (3) **Familial genetic counseling**. Understanding the molecular cause of the patients greatly facilitated further genetic counseling, including follow-up reproductive planning and screening of siblings. Case 041 was diagnosed with glycogen storage disease II. His sister was later diagnosed, started on enzyme replacement therapy, and lives a normal life at present.

After receiving a definite diagnosis, some patients controlled their symptoms through drugs or diet and avoided ineffective treatment; through systemic assessment and regular monitoring of the disease state, some patients could be intervened in time to avoid life-threatening diseases, such as metabolic crisis; while some parents decided to withdraw the life support equipment. All of the above scenarios reduced the medical care costs from different aspects.

The limitation of this study is that the turnaround time of WES is relatively long. At present, some studies have reported the application of rapid WES in critically ill children (Carey et al., 2020; Freed et al., 2020; Wang et al., 2020b). In future works, we will continue to improve testing methods, optimize testing procedures, and reduce testing costs.

## Conclusion

A retrospective analysis was performed on the clinical application of WES in a single PICU of China in the past 3 years. Approximately a quarter of critically ill children with suspected monogenic diseases were diagnosed by WES. In addition, 70% of the patients had a positive impact on medical treatment and families. WES played a key role in family decision-making and clinical treatment, and may improve the prognosis of some children and reduce the economic burden on families and the society. Therefore, we recommend that critically ill children with suspected monogenic diseases in PICUs be tested for WES. With the optimization of the testing process and the reduction of the cost, an increasing number of patients in PICUs are expected to benefit from WES.

## Data Availability Statement

According to national legislation/guidelines, specifically the Administrative Regulations of the People’s Republic of China on Human Genetic Resources (http://www.gov.cn/zhengce/content/2019-06/10/content_5398829.htm, http://english.www.gov.cn/policies/latest_releases/2019/06/10/content_281476708945462.htm), no additional raw data is available at this time. Data of this project can be accessed after an approval application to the China National Genebank (CNGB, https://db.cngb.org/cnsa/). Please refer to https://db.cngb.org/, or email: CNGBdb@cngb.org for detailed application guidance. The accession code HRA001063 should be included in the application.

## Ethics Statement

The studies involving human participants were reviewed and approved by the Institutional Review Board of the Beijing Children’s Hospital. Written informed consent to participate in this study was provided by the participants’ legal guardian/next of kin.

## Author Contributions

YL designed the analysis plan, collected and analyzed the data, drafted the manuscript, and critically reviewed and revised the manuscript for important intellectual content. CH made substantial contributions to the design of the study, coordinated and supervised the data collection, and critically reviewed and revised the manuscript for important intellectual content. XH, RG, JG, and ZQ led the analysis of whole exome sequencing, interpreted results, and critically reviewed and revised the manuscript for important intellectual content. KL, HG, JZ, JL, ZL, and XJ led the clinical treatment of the patients, interpreted the results, and critically reviewed and revised the manuscript for important intellectual content. WL and SQ conceptualized and designed the study, coordinated and supervised the data collection, and critically reviewed the manuscript for important intellectual content. All authors approved the final manuscript as submitted and agreed to be accountable for all aspects of the work.

## Conflict of Interest

The authors declare that the research was conducted in the absence of any commercial or financial relationships that could be construed as a potential conflict of interest.

## Publisher’s Note

All claims expressed in this article are solely those of the authors and do not necessarily represent those of their affiliated organizations, or those of the publisher, the editors and the reviewers. Any product that may be evaluated in this article, or claim that may be made by its manufacturer, is not guaranteed or endorsed by the publisher.

## Footnotes

1
https://omim.org/

2
https://geneyx.com/geneyxanalysis/

3
https://hpo.jax.org/app/
